# DC bias immune nanocrystalline magnetic cores made of Fe_73_Nb_3_Cu_1_B_7_Si_16_ ribbon with induced transverse magnetic anisotropy

**DOI:** 10.1186/s11671-016-1279-y

**Published:** 2016-02-05

**Authors:** Anton Nosenko, Olexandr Rudenko, Taras Mika, Igor Yevlash, Olexandr Semyrga, Viktor Nosenko

**Affiliations:** G.V. Kurdyumov Institute for Metal Physics of National Academy of Sciences of Ukraine, 6, Academician Vernadsky Boulevard, Kyiv, 03142 Ukraine

**Keywords:** Nanocrystalline magnetic core, Induced magnetic anisotropy, Tensile stress, DC bias immunity

## Abstract

The comparative analysis of magnetic properties of cut cores made of nanocrystalline Fe_73_Nb_3_Cu_1_B_7_Si_16_ alloy ribbon and cores made of the same ribbon with preliminary tension-induced transverse magnetic anisotropy was carried out. The possibility of improving magnetic properties of cut cores, decreasing loss, and increasing DC bias immunity of reversible magnetic permeability is presented. The influence of induced magnetic anisotropy on DC bias immunity of reversible magnetic permeability was investigated. The advantages and disadvantages of new cores (made of ribbon heated under tensile stress) over cut ones were determined.

## Background

Soft magnetic alloys are used in magnetic cores of various inductive components (of transformers and chokes) [1, 2]. Magnetic cores made of nanocrystalline Fe–Nb–Cu–B–Si system alloys occupy the leading position due to their high initial magnetic permeability and low remagnetization loss [3]. Volume fraction of α-Fe(Si) nanocrystals in them is 75–80 %; their size is about 10–12 nm [3–6]. Excellent soft magnetic properties of these alloys are explained by strong magnetic exchange interaction between α-Fe(Si) nanocrystals through remaining amorphous matrix [6].

Cut cores made of nanocrystalline Fe–Nb–Cu–B–Si alloy with linear loop are widely used in power electronics for the manufacture of linear and storage chokes as well as power reactors because these cores have lower remagnetization loss compared to crystalline ferrite cores and cut cores made of iron-based amorphous alloy [7]. It is known that the increase in nonmagnetic gap length of cut cores made of Fe–Nb–Cu–B–Si alloy leads to effective magnetic permeability decrease and, unfortunately, to core loss increase [7]. Another disadvantage of cut cores is fringing effect (effective area of generated magnetic lines flux in nonmagnetic gap is noticeably larger than core cross section area) which in electric circuit can negatively influence neighboring elements of electronic board.

It is known that for uncut cores made of Fe–Nb–Cu–B–Si alloy, high remagnetization loop linearity can be obtained by inducing uniaxial transverse magnetic anisotropy in them using magnetic field annealing [8, 9] and/or annealing under tensile stress [10–12]. Ribbon annealed under tensile stress has higher induced transverse magnetic anisotropy than the one annealed in transverse magnetic field [13]. The main contribution to the induced magnetic anisotropy originates from the residual deformation of lattice of nanosized crystals of α-Fe(Si) solid solution ordered by DO_3_ type [14] and the magnetoelastic anisotropy of Fe-enriched grains due to tensile back stresses exerted by inelastically deformed amorphous matrix [11]. It is known that such ribbon can be annealed under very high tensile stress up to 800 MPa [13].

Cores (made of Fe–Nb–Cu–B–Si alloy with tension-induced transverse magnetic anisotropy) have a number of advantages compared to cores made of other alloys, which are characterized by the same magnetic permeability. The main advantages are the following: high-frequency stability of magnetic permeability [12, 15–18] and low core loss in frequency range of widest use (1–100 kHz) [12, 15–19]. These cores are characterized by significantly higher remagnetization loop linearity compared to cut cores made of the same alloy and consequently by magnetic permeability independence on field strength [18].

DC bias on transformer primary winding presents in the most simple and widespread single-step converters; therefore, magnetic properties of cores used in them should be immune to such influence. It is known from literature that new cores have high immunity of magnetic permeability and core loss to DC bias field [16, 17]; however, the influence of induced magnetic anisotropy on DC bias immunity remains insufficiently investigated.

The aim of this work is to compare the main magnetic characteristics of cut cores made of nanocrystalline Fe_73_Nb_3_Cu_1_B_7_Si_16_ alloy ribbon with new cores made of the same ribbon subjected to electric current heating under tensile stress (up to 180 MPa) and to determine the main advantages of new uncut cores with induced transverse magnetic anisotropy.

## Methods

### Obtaining of amorphous ribbon and manufacture of nanocrystalline magnetic cores

The initial alloy was prepared in an experimental facility for induction melting in pure argon atmosphere. The alloy components (iron recovered in hydrogen (99.96 mass %), single-crystal silicon (99.999 mass %), niobium (≥99.7 mass %), Fe_2_B master alloy preliminary melted using amorphous boron (99.8 mass %), and electrical copper (99.9 mass %) were melted in a ceramic crucible and held 6–7 min at temperature 1500 °С; then, the melt was cast into a graphite mold. Thus, the ingot weighed about 1 kg was obtained, which was portioned for the following manufacture of amorphous ribbon in a facility for rapid quenching of the melt (RQM).

The amorphous ribbon was obtained in the available RQM open-type facility. Melting of the initial alloy was performed in a ceramic ampoule in a heat-resistant holder of precise motion system. After overheating of melt 150 °С above liquidus temperature *Т*_L_, it was ejected by an excess argon pressure of 20 kPa through a narrow nozzle of 0.4 × 10 mm from the distance of 0.2 mm maintained constant during the whole casting cycle.

The special designed chromium copper quenching disc of diameter about 580 mm was used in the facility. The linear rotary disc speed was 25 m/s. Formed ribbon was separated from the disc surface by special pneumatic “knife-remover” after 1/4 disc revolution, i.e., after 500 mm.

**Fig. 1:**
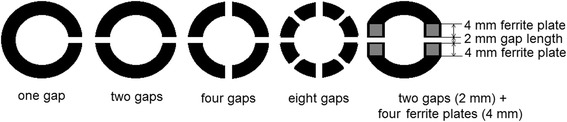
Schematic view of studied cut cores with nonmagnetic gaps

Toroidal cores with inner/outer diameter ratio 30/42 were obtained using electro-mechanical winding of initial amorphous ribbon and were annealed at temperature 550 °C for 1.5 h in Не atmosphere that ensured formation of nanocrystalline structure in them [3–6]. After annealing, the cores were monolithed by organosilicon lacquer and cut with one, two, four, or eight equal gaps (Fig. 1). Surfaces after cutting were polished on a grinding machine. Lengths of nonmagnetic gaps between core pieces were equal, and their total length was equal to total nonmagnetic gap length.

Core size with ferrite plates was the same as cut cores—30 × 42 mm, segments were equal to ferrite plates size—16 mm, and nonmagnetic gap length 4 mm were cut from the core (Fig. 1).

Amorphous ribbon crystallized under fast heating at simultaneous application of tensile stresses from 0 to 180 MPa along ribbon axis [18]. Fast heating was realized by conducting of electric current with density *j*_*h*_ = 42 A/mm^2^ and frequency 50 Hz through straight piece of the ribbon for *t*_*h*_ = 3.7 s that ensured its heating above 600 °С. We determined [18] that these heating modes (*j*_*h*_ = 42 А/mm^2^, *t*_*h*_ = 3.7 s) of amorphous ribbon allow to reach the maximum improvement of magnetic characteristics of cores made of the heated ribbon: initial magnetic permeability increase and core loss decrease. The reason of improving the magnetic characteristics is the formation (the time 3.7 s) of optimal volume fraction of α-Fe(Si) nanocrystals with minimal size in the amorphous matrix phase. Increasing time of heating by electric current leads to noticeable increase in the ribbon brittleness and deterioration of magnetic properties which is likely due to the formation of larger nanocrystals and possible negative influence of surface oxidation.

After heating, the ribbon was wound to form cores with inner/outer diameter ratio—30/42. Relatively large core diameter was selected to decrease the tension that appears in the ribbon after winding. It is known [16, 17] that core loss increases considerably when the inner diameter of the core is less than critical diameter (*D*_in_ ≤ *D*_c_).

**Fig. 2:**
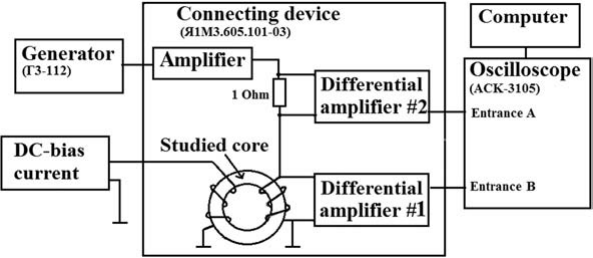
Functional scheme of MS-02 Universal LZQ Meter measuring complex

Investigation methods:

1. Initial magnetic permeability *μ*_i1_ of magnetic cores (at remagnetization frequency *f* = 1 kHz) was calculated by values of inductance of a few-turn coil in AC field 0.2 А/m measured by LCR Measurement Bridge HM8118 (HAMEG Instruments, Mainhausen, Germany).
2. Сore loss at different frequencies was measured using the measuring complex MS-02 B-H ANALYZER (MSTATOR, Novgorodskaya oblast, Russia) whose detailed description is presented in ref. [18].
3. Reversible magnetic permeability *μ*_rev1_ immunity to DC bias in magnetic field 2 A/m was measured using the measuring complex MS-02 Universal LZQ Meter (MSTATOR, Novgorodskaya oblast, Russia), whose functional scheme is presented in Fig. 2. AC current of frequency 1 kHz proportional to voltage on the instrument shunt of 1 Ohm is applied to the magnetizing coil from the broadband power amplifier. Regulated DC current is supplied to secondary winding of studied magnetic core. Measuring device has two precise differential amplifiers no.1 and no.2 which, correspondingly, amplify voltage signal from measuring-magnetizing coil and voltage signal from the instrument shunt connected to magnetization circuit. Signals from different amplifiers are registered at inputs B and A, respectively, of virtual digital two-channel storage oscilloscope ASK-3105 integrated in PC system unit.

Special software allows to record remagnetization loop and to determine its main characteristics: inductance factor (*А*_*L*_ = *L*/*N*^2^, where *L* is the inductance, *N* is the number of winding turns) of the investigated core at DC bias and, correspondingly, reversible magnetic permeability *μ*_rev_ are determined:

$$ {\mu}_{\mathrm{rev}}={A}_L\times {l}_c/\left({\mu}_0\times {A}_c\right), $$

where *l*_*с*_ is the core midline length, *A*_*c*_ is the effective cross section area of magnetic core, *N* is the number of winding turns, and *μ*_*0*_ is the magnetic constant.

## Results and discussion

**Fig. 3:**
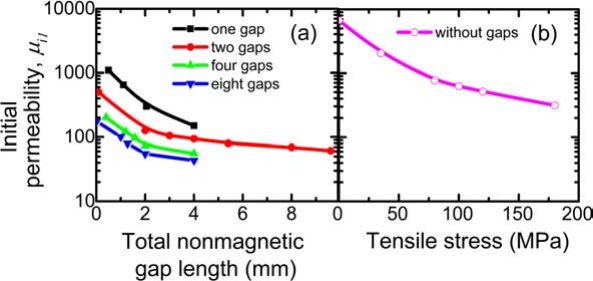
Dependence of initial magnetic permeability of cores on **a** total nonmagnetic gap length and **b** tensile stress

Figure 3a presents the dependence of initial magnetic permeability *μ*_i1_ on the total nonmagnetic gap length for cut cores with one, two, four, and eight gaps. It is obvious that *μ*_i1_ decreases with nonmagnetic gap length increase. Such behavior is associated with the magnetic resistance of the core. It can be seen also in Fig. 3a that the increase of gap number in cut core leads to considerable decrease of the initial magnetic permeability; this is related to the uniform distribution of magnetic resistances over the core.

It is also possible to decrease magnetic permeability *μ*_i1_ of the core made of the same alloy without cuts by the increase of tensile stresses applied to the ribbon during heating (Fig. 3b) [18] that is explained by inducing uniaxial transverse magnetic anisotropy in the ribbon [10].

One of the disadvantages of obtaining new cores by this method is the impossibility of decreasing magnetic permeability below 300 because brittle nanocrystalline ribbon breaks at the increase of tensile stresses.

**Fig. 4:**
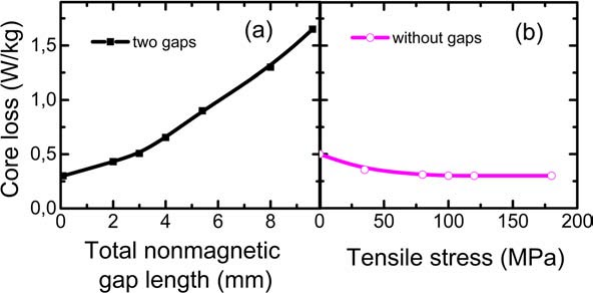
Dependence of core loss on **а** total nonmagnetic gap length and **b** tensile stress

Figure 4 presents dependences of core loss for cut and new cores at maximum magnetic induction *B*_*m*_ = 0.2 T and frequency *f* = 1 kHz. It can be seen that core loss for cut core (with two cuts) increases with nonmagnetic gap length increase (Fig. 4a), whereas core loss for new core decreases at tensile stress increase (Fig. 4b). Core loss decrease at tensile stress increase was already noted in ref. [18].

**Fig. 5:**
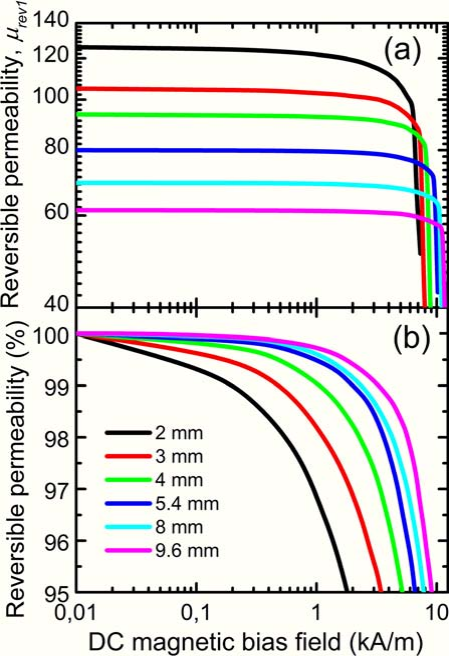
Dependences of reversible magnetic permeability (**a**) and relative change of reversible magnetic permeability (**b**) on DC bias field of cut cores with two gaps

Figure 5 shows the dependence of reversible magnetic permeability *μ*_rev1_ on DC bias field. It is obvious that the immunity of reversible magnetic permeability to DC bias of cut cores with two cuts can be enhanced substantially by the increase of total nonmagnetic gap length.

**Fig. 6:**
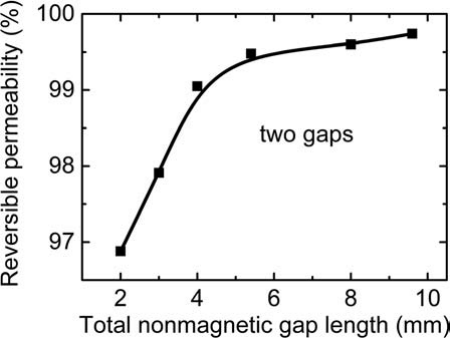
Dependence of reversible magnetic permeability at DC bias 1 kA/m on the total nonmagnetic gap length in cut core

Stability of magnetic permeability of cut cores was determined at DC bias 1 kA/m because cores in electric alternating current circuit work more often at such DC bias value. Figure 6 shows the dependence of relative decrease of magnetic permeability at DC bias 1 kA/m on the total nonmagnetic gap length in cut core. It can be seen that magnetic permeability stability increases steeply with total gap increase from 2 to 4 mm. Much weaker rise of magnetic permeability stability is observed at a further increase of nonmagnetic gap length from 4 to 9.6 mm. Taking into account that core loss rises at nonmagnetic gap length increase (Fig. 3a), we can draw the conclusion that total nonmagnetic gap 4 mm is optimal for obtaining high magnetic permeability stability and relatively low core loss.

**Fig. 7:**
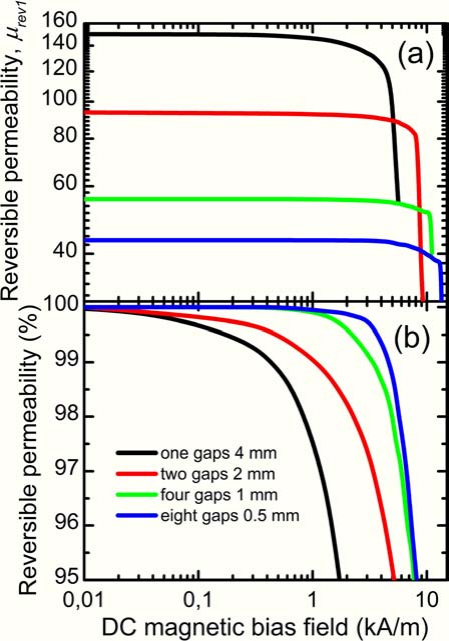
Dependences of reversible magnetic permeability (**a**) and relative change of reversible magnetic permeability (**b**) on DC bias field of cut cores with distributed total gap 4 mm

In order to enhance magnetic permeability stability in cut core, we have distributed the total gap of 4 mm over the core, one core was cut in four equal parts, and another one in eight parts. The total nonmagnetic gap length did not change, so core loss remained about the same as in the core cut in two parts with total nonmagnetic gap length 4 mm (Fig. 3a). Figure 7 presents experimental dependences of magnetic permeability on DC bias of cut cores with total nonmagnetic gap length 4 mm. Evidently, magnetic permeability stability increases considerably with gap number increase in cut cores; this is caused by the increase of magnetic flux uniformity in the core.

It can be seen in Fig. 7b that magnetic permeability is constant at DC bias 1 kA/m in cores with four and eight gaps. So the conclusion can be drawn that it is not reasonable to increase the gap number over four.

**Fig. 8:**
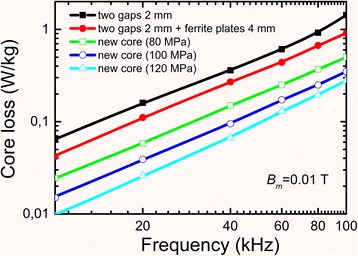
Frequency dependences of core loss for cut core and new core

Loss in the core that cut in two parts with the total gap length 4 mm can be decreased by attaching 4-mm-thick ferrite plates to gap surfaces, as described in ref. [7]. Dependence of core loss on remagnetization frequency of cut cores at *B*_*m*_ = 0.01 T is presented in Fig. 8. It is clear that cores with ferrite plates have lower loss compared to cut cores with the same nonmagnetic gap length 4 mm. However, we tried to decrease cut core loss, but it was always higher than that in new cores made of the ribbon with transverse anisotropy (electrically heated under tensile stress 80–120 MPa) (Fig. 8). It can be seen that new nanocrystalline cores are characterized by minor loss: two to four times less than cut core loss.

**Fig. 9:**
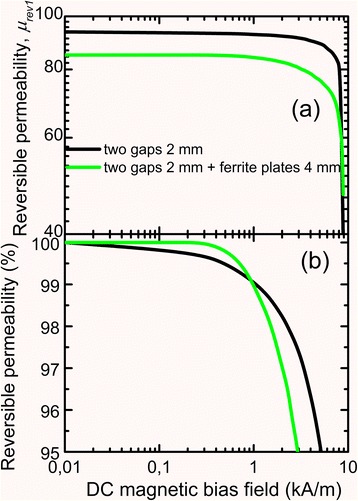
Dependences of reversible magnetic permeability (**a**) and relative change of reversible magnetic permeability (**b**) on DC bias field of cut cores without and with ferrite plates

Figure 9 presents experimental dependences of reversible magnetic permeability on DC bias for cores without and with 4-mm ferrite plates. It is clear that cores with ferrite plates and without them have the same stability of magnetic permeability to DC bias field 1 kA/m; at field strength >1 kA/m, the stability of magnetic permeability of cores with ferrite plates is slightly lower.

**Fig. 10:**
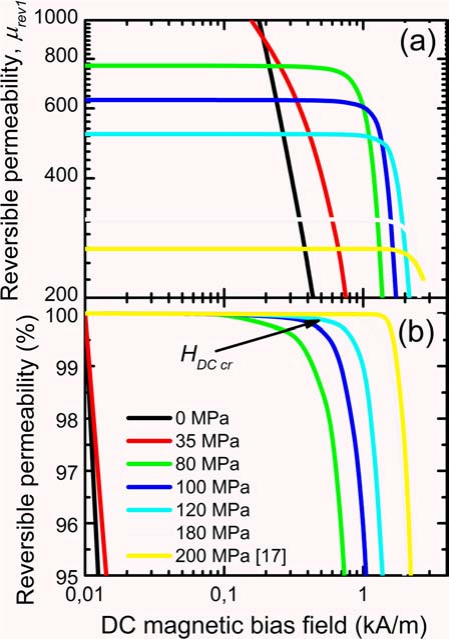
Dependences of reversible magnetic permeability (**a**) and relative change of reversible magnetic permeability (**b**) on DC bias field of new cores

Figure 10 presents experimental dependences of magnetic permeability on DC bias of new cores and analogous dependence of a core made of a ribbon annealed at 200 MPa taken from ref. [17]. DC bias immunity of *μ*_rev1_ increases substantially at the increase of tensile stresses applied to the ribbon during heating (nanocrystallization).

**Fig. 11:**
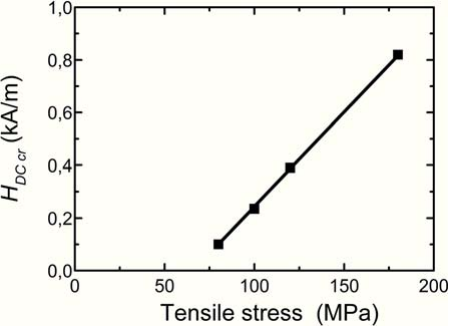
Dependence of DC bias critical field *H*_DC cr_ for new cores on tensile stress value (from 80 to 180 MPa)

Reversible magnetic permeability of new cores is constant up to DC bias critical field *H*_DC cr_, what is marked by arrow for the core made of the ribbon heated under tensile stress 120 MPa in Fig. 10b. Figure 11 shows that tensile stress increase (from 80 to 180 MPa) during heating of the ribbon (nanocrystallization) leads to linear increase of DC bias (*H*_DC cr_ increase) immunity of reversible magnetic permeability of new cores.

**Fig. 12:**
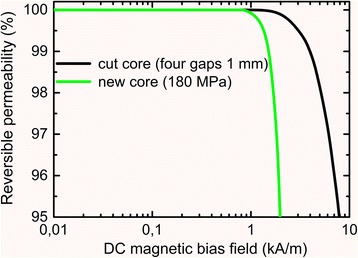
DC bias field dependences of relative change of reversible magnetic permeability of the cut core (with four cuts 1 mm each) and the new core (180 MPa)

Figure 11 presents experimental DC bias dependences of reversible magnetic permeability of the new core (made of the ribbon heated under tensile stress 180 MPa) and the cut core with four gaps 1 mm each. It can be seen that both cores have the same 1 kA/m DC bias immunity values of magnetic permeability. However, at high DC bias (up to 8 kA/m and higher), cut core has no alternative: its permeability decrease by 5 % only (Fig. 12) is permissible for majority cases of application in power electronics.

Taking into account that DC bias does not exceed 1 kA/m under commonly used conditions of core exploitation, we can state that new core (made of the ribbon crystallized under application of tensile stress 180 MPa) have significant advantage by loss level over cut cores made of the same Fe_73_Nb_3_Cu_1_B_7_Si_16_ alloy.

## Conclusions

1. Reversible magnetic permeability can be controllably decreased for cut cores and nanocrystalline cores by the increase of nonmagnetic gap length and/or tensile stress during heating (nanocrystallization) of as-cast amorphous ribbon before manufacture (winding) of new toroidal cores. Permeability decrease is accompanied by core loss increase for cut core and core loss decrease for new nanocrystalline core.
2. Distribution of 4 mm total gap over the core leads to higher DC bias immunity of magnetic permeability.
3. Increase of tensile stress (from 80 to 180 MPa) applied to the ribbon during its heating leads to linear increase of DC bias immunity of reversible magnetic permeability of new nanocrystalline cores.
4. New nanocrystalline cores (made of the ribbon heated under tensile stress up to 180 MPa) and cut cores (with four cuts 1 mm each) made of the same Fe_73_Nb_3_Cu_1_B_7_Si_16_ alloy have constant magnetic permeability up to 1 kA/m DC bias field. Wherein, new cores are characterized by very low loss: two to four times less than cut core loss.

The obtained characteristics are advantageous for application of new magnetic cores made of Fe_73_Nb_3_Cu_1_B_7_Si_16_ alloy in power reactors and line chokes of filters of switch mode power supplies.
